# Genomic analysis of *qnr*-harbouring IncX plasmids and their transferability within different hosts under induced stress

**DOI:** 10.1186/s12866-022-02546-6

**Published:** 2022-05-19

**Authors:** Tomas Nohejl, Adam Valcek, Ivo Papousek, Jana Palkovicova, Alexander M. Wailan, Hana Pratova, Marco Minoia, Monika Dolejska

**Affiliations:** 1 Department of Biology and Wildlife Diseases, Faculty of Veterinary Hygiene and Ecology, University of Veterinary Sciences Brno, Brno, Czech Republic; 2 CEITEC, University of Veterinary Sciences Brno, Brno, Czech Republic; 3 Faculty of Medicine, Biomedical Center, Charles University, Pilsen, Czech Republic; 4 Parasites and Microbes, Wellcome Trust Sanger Institute, Wellcome Genome Campus, Hinxton, CB10 1SA UK; 5 Department of Clinical Microbiology and Immunology, Institute of Laboratory Medicine, The University Hospital Brno, Brno, Czech Republic

**Keywords:** IncX, IS*26*, PMQR, Qnr, Plasmid conjugation, Transfer rate, *Escherichia coli*, Induced stress, Bacterial background, Plasmid persistence

## Abstract

**Background:**

Conjugative plasmids play a major role in the dissemination of antibiotic resistance genes. Knowledge of the plasmid characteristics and behaviour can allow development of control strategies. Here we focus on the IncX group of plasmids carrying genes conferring quinolone resistance (PMQR), reporting their transfer and persistence within host bacteria of various genotypes under distinct conditions and levels of induced stress in form of temperature change and various concentrations of ciprofloxacin supplementation.

**Methods:**

Complete nucleotide sequences were determined for eight *qnr*-carrying IncX-type plasmids, of IncX1 (3), IncX2 (3) and a hybrid IncX1-2 (2) types, recovered from *Escherichia coli* of various origins. This data was compared with further complete sequences of IncX1 and IncX2 plasmids carrying *qnr* genes (*n* = 41) retrieved from GenBank and phylogenetic tree was constructed. Representatives of IncX1 (pHP2) and IncX2 (p194) and their *qnrS* knockout mutants, were studied for influence of induced stress and genetic background on conjugative transfer and maintenance.

**Results:**

A high level of IncX core-genome similarity was found in plasmids of animal, environmental and clinical origin. Significant differences were found between the individual IncX plasmids, with IncX1 subgroup plasmids showing higher conjugative transfer rates than IncX2 plasmids. Knockout of *qnr* modified transfer frequency of both plasmids. Two stresses applied simultaneously were needed to affect transfer rate of wildtype plasmids, whereas a single stress was sufficient to affect the IncX *ΔqnrS* plasmids. The conjugative transfer was shown to be biased towards the host phylogenetic proximity. A long-term cultivation experiment pointed out the persistence of IncX plasmids in the antibiotic-free environment.

**Conclusions:**

The study indicated the stimulating effect of ciprofloxacin supplementation on the plasmid transfer that can be nullified by the carriage of a single PMQR gene. The findings present the significant properties and behaviour of IncX plasmids carrying antibiotic resistance genes that are likely to play a role in their dissemination and stability in bacterial populations.

**Supplementary Information:**

The online version contains supplementary material available at 10.1186/s12866-022-02546-6.

## Introduction

The continuous spread of antimicrobial resistance (AMR) represents one of the world's most pressing public health problems. Recent surveillance studies demonstrate that resistance rates continue to increase, particularly, the emergence of quinolone resistance rises exponentially within *Escherichia coli* infections [1, 2]. Horizontal gene transfer of mobile genetic elements, especially plasmids, plays a major role in the dissemination of AMR [3]. Various plasmid-mediated quinolone resistance (PMQR) genes including different *qnr* families (*qnrA*, *qnrB*, *qnrC*, *qnrD, qnrE, qnrS and qnrVC*), *aac(6′)-Ib-cr*, *crpP* and genes encoding quinolone efflux pumps (*qepA*, *oqxAB* and *qacBIII*) have been described [4]. The PMQR genes are harboured by a broad spectrum of plasmids including IncX-type plasmids [5].

IncX plasmids have a narrow spectrum of hosts (*E. coli*, *Pseudomonas aeruginosa*, *Klebsiella pneumoniae, Salmonella enterica*) and currently have eight subgroups (IncX1—IncX8) identified via replicon typing [6]. This incompatibility group of plasmids contains iterons and core genes of the backbone which are responsible for replication (*pir*), partitioning (*par*), pilus synthesis (*pil*), conjugation (*tax*) [6]. Apart from PMQR, a great variety of other AMR mechanisms such as extended-spectrum β-lactamases (ESBL), AmpC-type β-lactamases, carbapenemases and more recently colistin resistance were described to be linked with this plasmid family [7].

The spread of AMR within the bacterial populations is predominantly mediated by conjugation and is known to be affected by antibiotic supplementation causing SOS response and overexpression of certain genes [8–10]. Conjugation rate can also be altered not only by antibiotics but by the physiological state of bacterial cells before conjugation. Therefore, the effect of temperature change to lower temperatures is assumed to have influence on the transfer rate [11]. Lower temperatures habitually occur in environmental reservoirs of antibiotic resistant bacteria, such as wastewater.

Many other aspects may play a distinct role in the complex system of the conjugative process. Recent studies suggested that bacterial hosts of similar phylogenetic background may significantly promote plasmid transfer rate and thus directly enhance the spread of antibiotic resistance within the bacterial population [12]. In addition plasmid stability is known to play an integral part in the overall success of plasmid transfer and the rampant spread of AMR [13]. The effects of the host’s internal environment on the plasmid transfer regulation also contribute, however only a few plasmids of limited clinical interest have been studied [14].

The investigation presented here is a follow-up study on the previous work of Dobiasova and Dolejska (2016) in which we identified several IncX1 and IncX2 plasmid sublineages widely distributed in non-related *E. coli* from various sources and geographical areas. The aim of this investigation was to further understand the factors which influence their successful transfer through a series of conjugation assays under various conditions complemented by the detailed genomic analysis of complete nucleotide sequences of various IncX sublineages plasmids via whole genomic sequencing.

## Materials and methods

### IncX plasmids

The eight plasmids from IncX1 and IncX2 subgroups carrying *qnr* gene originated from a diverse set of *E. coli* isolates of various sources (Table 1). These plasmids were subjected to sequencing and complete plasmid structure reconstruction. Plasmid selection was based on the data obtained by Dobiasova and Dolejska (2016) and representatives of the dominant plasmid lineages were detected in *E. coli* isolates. One representative plasmid from each subgroup, pHP2 from IncX1 and p194 from IncX2, were further used for mating assays and plasmid stability experiments.

**Table 1:** Characteristics of eight IncX plasmids recovered from various sources

Plasmid	Inc	PMQR	ARG	Source	Country^a^	Size (bp)	GenBank no	Frequency of transfer^b^	Standard deviation
pHP2	X1	*qnrS1*	*bla*_TEM-1B_	Rook	CZ	47.686	MH121702	2.32 × 10^–2^	6.06 × 10^–3^
pCE780h4	X1	*qnrS1*	*bla*_TEM-1B_, *tet*(A), *floR*	WWTP^**c**^	CZ	60.848	MT859330	3.79 × 10^–2^	6.15 × 10^–3^
pCE1551	X1	*qnrS1*	*bla*_TEM-1B_, *sul3*, *floR*, *aadA1*	Gull	AUS	56.495	MT859328	3.62 × 10^–2^	1.23 × 10^–2^
pCE1594	X1-X2	*qnrS1*	*bla*_TEM-176_, *aph(3')-Ia*	Gull	AUS	43.897	MT859327	3.48 × 10^–7^	1.31 × 10^–7^
pHE40	X1-X2	*qnrS1*	*tet*(M), *bla*_TEM-1B_, *aadA2*	Rook	ES	45.484	MT859326	3.26 × 10^–7^	1.19 × 10^–7^
p194	X2	*qnrS1*	*tet*(A)	Mallard	CZ	39.584	MH121703	1.04 × 10^–4^	2.39 × 10^–5^
p615cip	X2	*qnrB19*	-	Dog	SK	31.584	MT859325	5.24 × 10^–4^	4.00 × 10^–4^
pHP103	X2	*qnrS2*	*dfrA14*	Rook	CZ	36.516	MT859324	3.53 × 10^–4^	1.78 × 10^–4^

^**a**^CZ stands for Czech Republic, AUS stands for Australia, ES stands for Spain and SK for Slovakia

^**b**^Frequency of transfer was calculated as the number of transconjugants per the number of donors (T/D)

^**c**^WWTP stands for wastewater treatment plant

### Plasmid sequencing

IncX plasmids were introduced into plasmid-free *Escherichia coli* TOP10 (Invitrogen™, USA) by chemical transformation and transformants were selected on Luria–Bertani (LB) agar (Difco, France) plates supplemented with ciprofloxacin (0.05 µg/mL). Plasmid transfer was confirmed by PCR assays for *qnrS* and *taxC* genes as well as by S1-nuclease pulsed-field gel electrophoresis. Plasmid DNA was extracted from corresponding single plasmid-carrying *E. coli* TOP10 transformants or original *E. coli* MT102 transconjugants obtained from previous study [15] using Invitrogen PureLink Plasmid Midiprep Kit (Thermo Fisher Scientific, Massachusetts). Plasmids pHP2 and p194 were sequenced by 454 pyrosequencing technology by Roche (Roche 454, Branford, USA). For the remaining plasmids, libraries were prepared using Nextera XT Library Preparation Kit (Illumina Inc., San Diego, CA, USA) according to manufacturer’s instructions and consequently 2 × 251 bp pair-end sequenced using Illumina MiSeq platform (Illumina Inc.). Moreover, plasmid pHP103 that failed to be closed by Illumina sequencing was also sequenced using MinION platform [Oxford Nanopore Technologies (ONT) Ltd., Oxford, England] using 1D Ligation Barcoding Kit and R9.4 flow cell in order to provide long reads as a scaffolding for consequent hybrid assembly.

### Data analysis and plasmid assembly

Plasmids sequenced on Roche’s 454 pyrosequencer were assembled automatically by the Roche Newbler assembly software 2.3 (Roche 454). The raw reads from Illumina MiSeq platform were low-quality (Q ≥ 20) ends and adaptor residues trimmed using Trimmomatic v0.36 [16] and assembled using SPAdes 3.11.0 [17] with “–careful” remark. The fast5 data from ONT MinION platform were base-called, demultiplexed and converted to fastq format using Albacore v2.0.2 (ONT) and adaptor trimmed using Porechop v0.2.2 [18]. The leftover chromosomal reads were filtered out by mapping the reads on the *E. coli* TOP10 chromosome using BWA-MEM algorithm [19, 20]. The fastq files and trimmed short reads of pHP103 were then used to perform a hybrid assembly using Unicycler v0.4.0 [21]. The short reads were then mapped to the resulting consensus and manually checked and corrected of indels. The overlaps confirming circular conformation of plasmids were verified using PCR and Sanger sequencing. For plasmids pHP2 and p194, which were sequenced by 454 pyrosequencing technology, homopolymer regions differing in length between pHP2 and plasmid 0228 (CP012734), and between p194 and RCS81_p (LT985317), respectively, were verified using PCR and Sanger sequencing. Complete circular sequences were annotated using Geneious software v9.0.5 (Biomatters, Auckland, New Zealand) in accordance with annotation style of previous studies [22].

### In silico and phylogenetic analyses

The Basic Local Alignment Search Tool (BLAST) search was performed (August 10, 2020) using Megablast option in Geneious v9.0.5 (Biomatters, Auckland, New Zealand) to search for complete plasmid sequences using alignment of IncX1 and IncX2 replicon sequences as a query. From the total of 208 sequences, 49 sequences (including 8 from this study) carrying *qnr* gene were selected based on the occurrence of *qnr* gene and IncX replicon. The open reading frames of the acquired sequences were predicted using Prokka v1.13 [23]. In order to determine the core- and pangenome, annotated gff files were then used as an input for multi-sequence alignment using Roary [24] with minimum percentage identity of 60%. A phylogenetic tree was constructed using GTRCAT model in RAxML v8.2.4 [25] and visualized in iTOL tool [26]. Comparisons of circular and linear plasmid sequences were performed using BLAST Ring Image Generator (BRIG v0.95) [27] and Clinker v0.0.13 [28], respectively.

### Frequency of IncX plasmid transfer

In order to determine the conjugation rate of IncX plasmids, each of all eight featured plasmids was transformed into *E. coli* TOP10 and transformants were used as donor cells for mating assays. Donor mid-exponential phase culture (OD_600_ = 0.7—0.8) and overnight culture of recipient *E. coli* A15 (OD_600_ > 1.5), 500 µl of each, were centrifuged at 5.000 rpm for 2 min and pellets were washed-out of antibiotics by LB broth. Donor and recipient cultures were mixed (ratio of cells 1:2) and centrifuged again and the pellet was resuspended in 50 µl of LB broth. Mixed cultures were co-incubated on sterile 0.22 µm bacteriological filter on LB agar plate for 1 h at 37 °C. Similarly, donor mid-exponential phase culture alone was simultaneously incubated and served as a control of donor growth. Donor cells were selected and colony counted on LB agar plates supplemented with ciprofloxacin (0.05 µg/mL). Transconjugants were selected and colony counted on LB agar plates supplemented with ciprofloxacin (0.05 µg/mL) and sodium azide (100 µg/mL). Mating assays were performed in triplicates. Transconjugant colonies (4 per sample) were confirmed by PCR assays for PMQR and *taxC* genes [29]. Frequency of plasmid transfer was calculated as the number of transconjugants per the number of donors (T/D). For statistical analysis the conjugation frequencies were tested with Student's T-test, and a p value < 0.05 was considered statistically significant.

### Preparation of plasmid mutants with knockout of *qnrS* gene

In order to determine the influence of *qnrS* gene on plasmid transfer, the pHP2 and p194 plasmids within *E. coli* TOP10 were subjected to *qnrS* gene knockout by 15 bp insertion using Mutation Generation System Kit with MuA Transposase (Thermo Fisher Scientific, Massachusetts) according to the manufacturer instructions. Created plasmid mutant libraries were additionally incubated on LB plates with ciprofloxacin (0.05 µg/mL) and on LB plates without antibiotics. Negative growth on LB plates with ciprofloxacin determined the knockout of *qnrS* gene. Plasmid mutants were confirmed by PCR assays as described above while mutants with knockout in *qnrS* gene were negative for *qnrS* gene and positive for *taxC* gene.

### Transferability of pHP2 and p194 and their *ΔqnrS* variants under stress

In order to determine the effect of environmental stress and occurrence of *qnrS* gene on frequency of plasmid transfer, the mating assays were conducted using the protocol as described above with changes in selection markers for *ΔqnrS* plasmid donors and their transconjugants due to their susceptibility to ciprofloxacin (Supplementary Table S1). Additionally, the plasmid transfer assays were conducted at 25 °C and 37 °C in combination with ciprofloxacin in concentration of 0, 0.001, 0.05, 0.5 and 2 µg/mL supplemented into the mating agar plates. Every viable combination of the conjugation assays was performed with strains carrying the plasmid and their *ΔqnrS* variants, therefore pHP2, pHP2 *ΔqnrS*, p194 and p194 *ΔqnrS. E. coli* TOP10 served as a donor whereas sodium azide resistant *E. coli* A15 served as a recipient. The experiment was performed all in accordance with the experimental design defined in the Supplementary Table S1.

### Effect of bacterial background divergence on plasmid transferability

Mating assays to determine the influence of bacterial background were conducted at 37 °C with *E. coli* ST10 (TOP10, A15) strains and *E. coli* ST127 (UPEC536) and *E. coli* ST131 strains serving variably as donors and recipients. Experiment was performed to examine the frequency of transfer between genetically closely and distantly related strains (Supplementary Table S2). Individual mating conditions with representative plasmids pHP2 and p194 are defined in the Supplementary Table S3.

In order to ensure uniform bacterial background used for the mating assays and to maintain proper detection, the recipient cells were marked with plasmid pBGC [30]. Plasmid pBGC carrying *gfp* and *catA1* genes was transferred to recipient *E. coli* A15, UPEC536 and ST131 cells via electroporation under the following conditions: 1.8 kV, 200 Ω, 25 µFar. Transformants were selected on LB agar with sodium azide (100 µg/mL) and chloramphenicol (30 µg/mL). Successful transformation was verified via detection of the *gfp* gene by PCR assay [30].

### Plasmid persistence

All strains of *E. coli* transformants and transconjugants with plasmids pHP2, pHP2 *ΔqnrS*, p194 or p194 *ΔqnrS* were inoculated into antibiotic-free LB broth and cultivated for five days in order to test their maintenance. The 50 µL of each culture strain were daily reinoculated into 50 mL of fresh LB media, serially diluted and plated on LB agar in order to acquire 100—200 colonies per plate. One hundred colonies were randomly selected from every plate and replica plated simultaneously onto antibiotic-free and ciprofloxacin-supplemented (0.05 µg/mL) LB agar plates. In order to detect strains with *ΔqnrS* mutant plasmids, ampicillin (100 µg/mL) or tetracycline (20 µg/mL) supplemented plates were used for pHP2 *ΔqnrS* or p194 *ΔqnrS*, respectively*.* Bacterial growth on the antibiotic-free plate but not on the antibiotic-supplemented plate would determine the loss of the plasmid of interest. All in accordance with the previously established protocol [31].

## Results

### Complete nucleotide sequences of IncX1 and IncX2 plasmids and their hybrids

The IncX1 and IncX2 plasmids were distinguished based on archetype plasmid R6K [32]. In this study, we describe complete plasmid sequences of three IncX1 and three IncX2 *qnr*-encoding plasmids. Moreover, we bring complete plasmid sequences of two IncX1-2 hybrid plasmids.

**Fig. 1:**
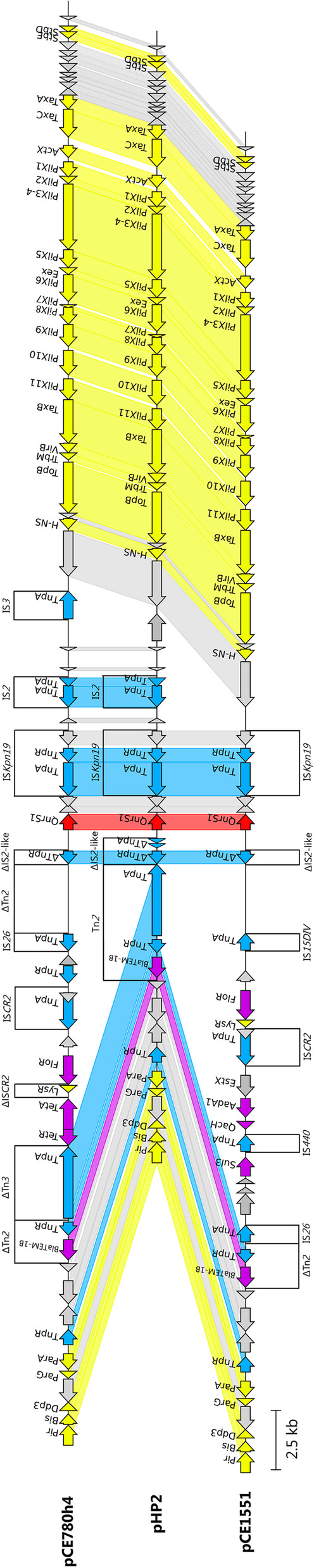
Structural comparison of IncX1 plasmids (pCE780h4, pHP2 and pCE1551). Yellow colour represents genes of plasmid backbone, blue colour represents transposons and insertion sequences, purple colour represents genes encoding antibiotic resistance, red colour represents *qnr* variant and grey colour represents accessory genes. Δ represents an incomplete gene or sequence. Vertical blocks between sequences indicate regions of shared identity above 95% according to blastn

**Fig. 2:**
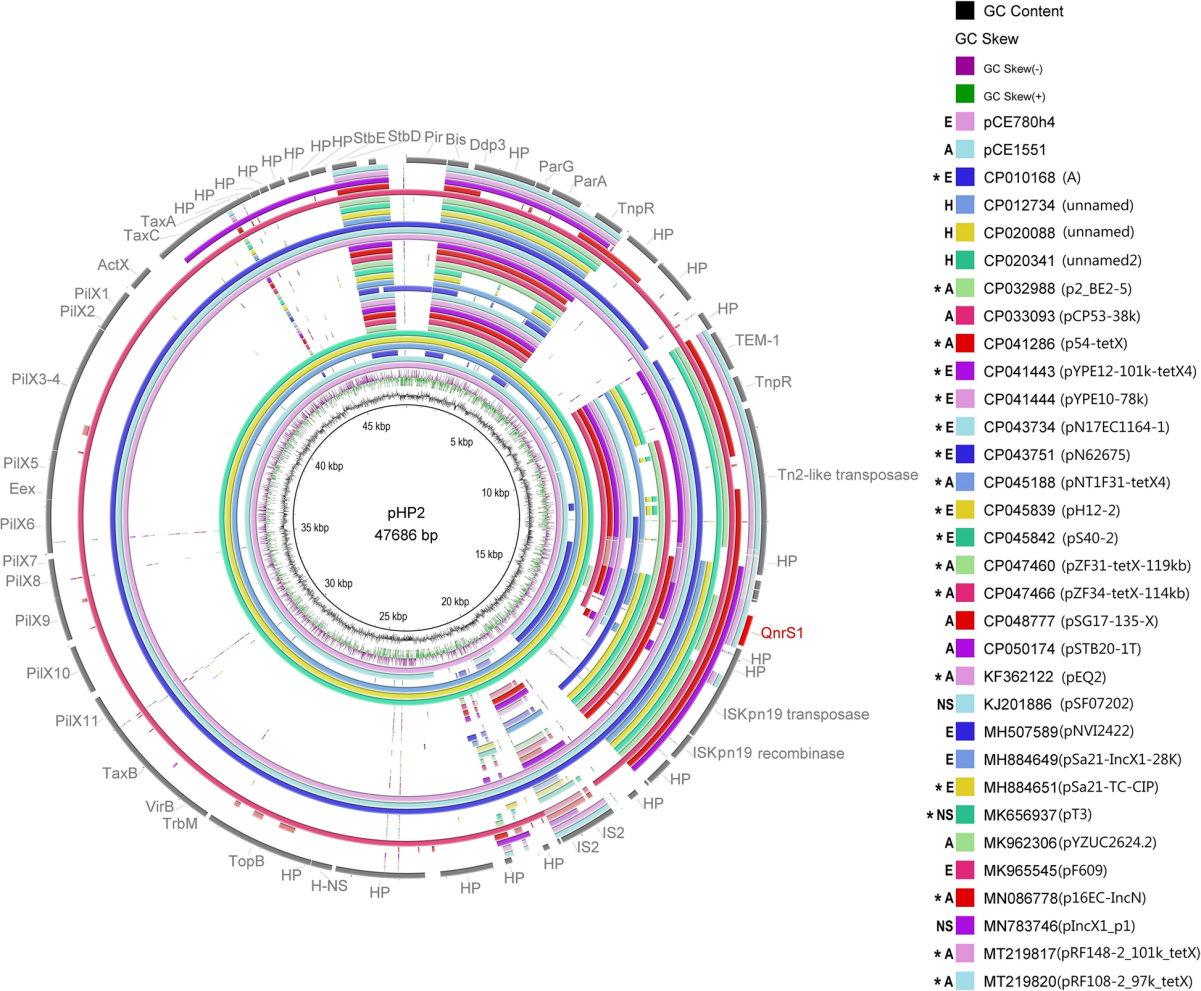
BRIG comparison of the IncX1 plasmids carrying *qnr* gene. Coloured circle segments show identical sequences of plasmids retrieved from GenBank (depicted from inside to outside) with plasmids from this study. IncX1 plasmid group included 17 multi-replicon plasmids (*) with other replicon types (Col, FI, HI1, I, N and R), resulting in their high sequence variability. H (human), A (animal), E (environmental) and NS (not specified) represent the plasmid source

IncX1 plasmids pHP2, pCE780h4 and pCE1551 shared well conserved backbone with a typical core gene set (*pir, bis, par, hns, topB, pilX, actX* and *taxA-C*) for replication, stability, and conjugal transfer (Fig. 1). They all carried the *qnrS1* gene associated with IS*Kpn19*. Plasmid pHP2 shared highest sequence identity (100% coverage and 99.98% identity) with plasmid 0228 (CP012734) originating from human *Shigella flexneri* isolate from China (Fig. 2). Plasmid pHP2 was also highly similar with plasmids found in human *S. flexneri* isolates from China (KJ201886 and CP020088) [33] and with pEQ2 (KF362122) plasmid of horse origin from the Czech Republic, which is a well described IncX1-HI1 fusion plasmid [34]. Plasmids pCE780h4 and pCE1551 shared highest coverage (83% and 76%, respectively) and identity (both 100%) with pHP2.

**Fig. 3:**
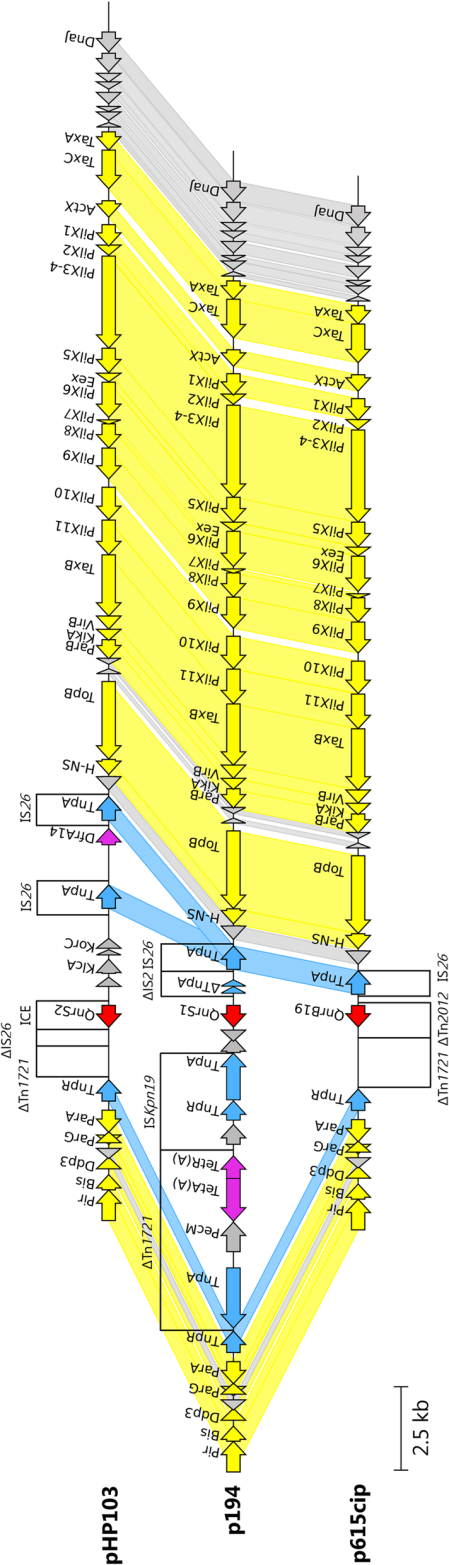
Structural comparison of IncX2 plasmids (pHP103, p194 and p615cip). Yellow colour represents genes of plasmid backbone, blue colour represents transposons and insertion sequences, purple colour represents genes encoding antibiotic resistance, red colour represents *qnr* variant and grey colour represents accessory genes. Δ represents an incomplete gene or sequence. Vertical blocks between sequences indicate regions of shared identity above 95% according to blastn

**Fig. 4:**
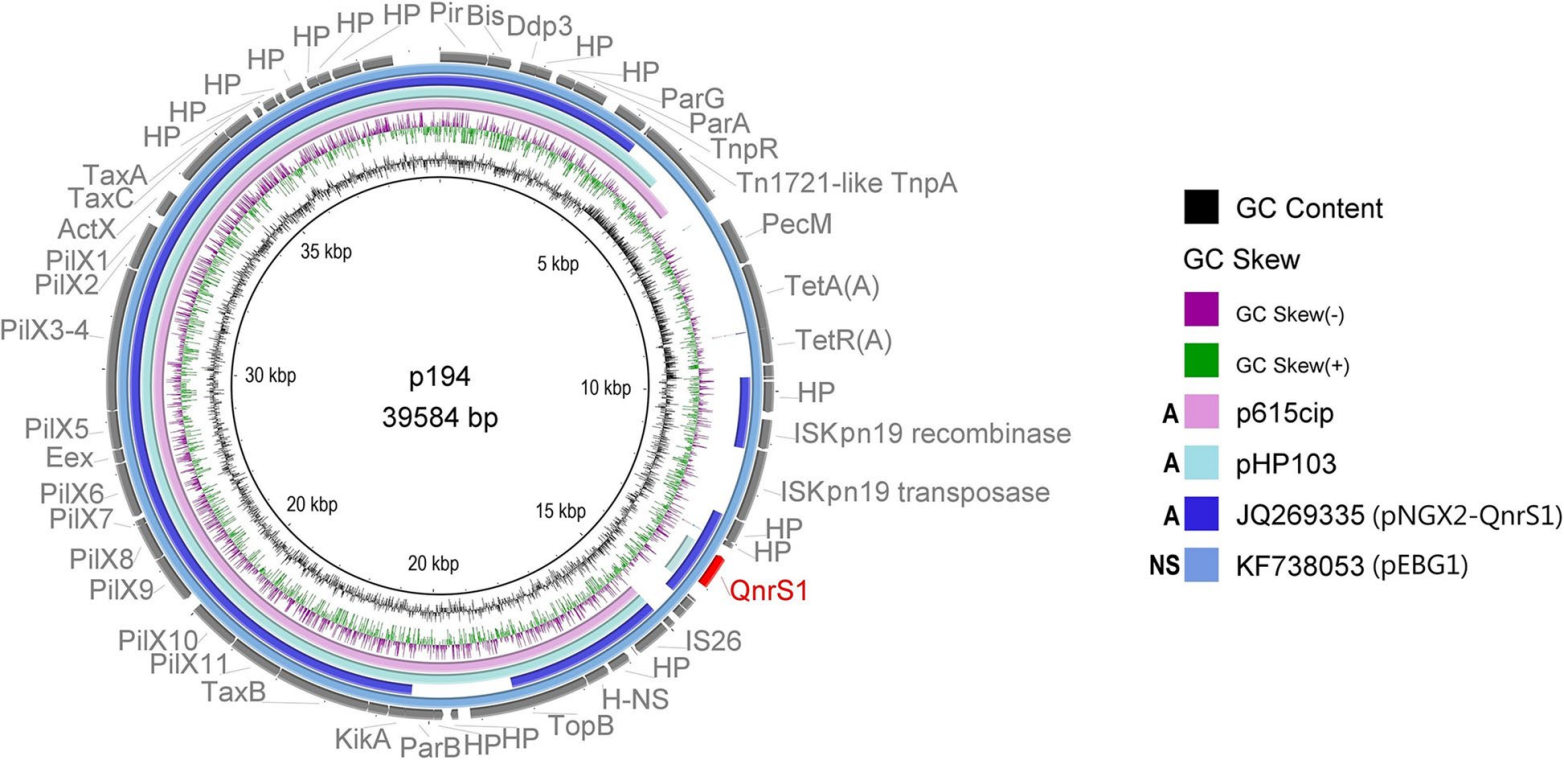
BRIG comparison of the IncX2 plasmids carrying *qnr *gene. Coloured circle segments show identical sequences of plasmids retrieved from GenBank (depicted from inside to outside) highly similar to the plasmids from this study. A (animal) and NS (not specified) represent the plasmid source

All IncX2 plasmids from this study had a typical backbone of R6K IncX2 plasmid [35] including genes for replication, partitioning, stability, maintenance, and DNA transfer. Plasmids p194, p615cip and pHP103 (Fig. 3) carried PMQR genes *qnrS1*, *qnrB19* and *qnrS2*, respectively, and unlike IncX1 group these genes were closely associated with IS*26*. Plasmids p615cip and pHP103 shared highest coverage (96% and 85%, respectively) and identity (99.98% and 99.99%, respectively) with p194. Plasmid p194 shared highest identity (84% coverage and 99.89% identity) with plasmid RCS81_p (LT985317) isolated in France and second highest (70% coverage and 99.79% identity) with IncX2 archetype plasmid R6K (Fig. 4).

**Fig. 5:**
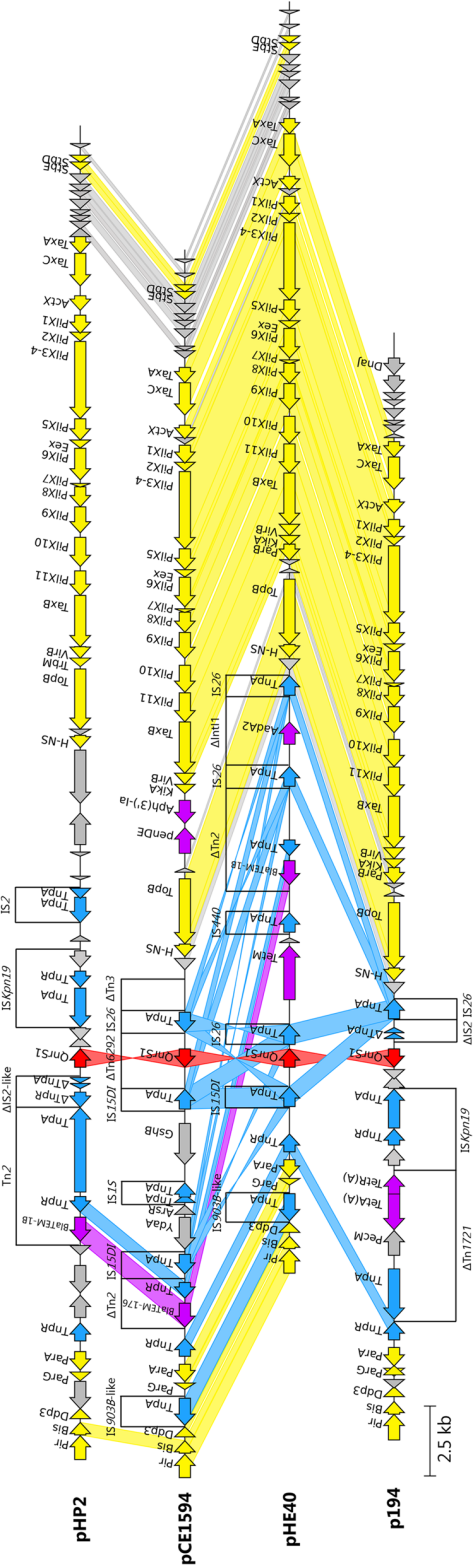
Structural comparison of IncX1-2 plasmids to IncX1 and IncX2 plasmids (pHP2, pCE1594, pHE40 and p194). Yellow colour represents genes of plasmid backbone, blue colour represents transposons and insertion sequences, purple colour represents genes encoding antibiotic resistance, red colour represents *qnr *variant and grey colour represents accessory genes. Δ represents an incomplete gene or sequence. Vertical blocks between sequences indicate regions of shared identity above 95% according to blastn

**Fig. 6:**
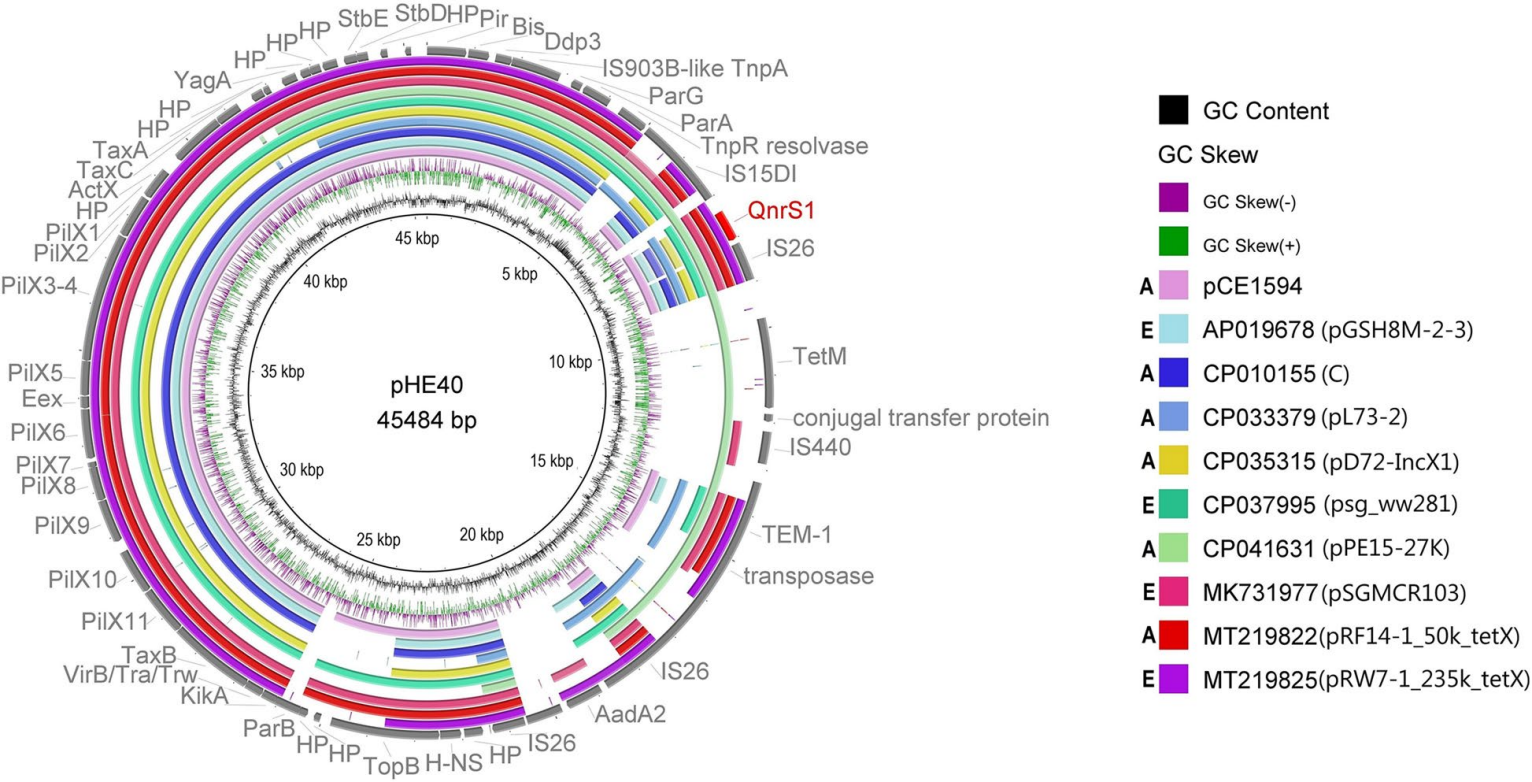
BRIG comparison of the IncX1-2 plasmids carrying *qnr* gene. Coloured circle segments show identical sequences of plasmids retrieved from GenBank (depicted from inside to outside) highly similar to the plasmids from this study. A (animal) and E (environmental) represent the plasmid source

Hybrid plasmids pCE1594 and pHE40 shared conserved backbone modules for replication, partitioning and stability from IncX1 plasmids and for maintenance and DNA transfer from IncX2 plasmids (Fig. 5). Both plasmids carried *qnrS1* flanked by IS*26* and IS*15DI* elements. pHE40 also shares a conserved backbone with other publicly available IncX2 plasmids (Fig. 6).

Apart from *qnr* genes, all plasmids with the exception of p615cip harboured other antibiotic resistance genes (ARG) (Table 1). The ARG on individual plasmids are associated with insertion sequences and transposons as depicted within figures of structural comparison of each mentioned group of plasmids.

### Phylogenetic analysis of IncX1 and IncX2 plasmids carrying *qnr* genes

**Fig. 7:**
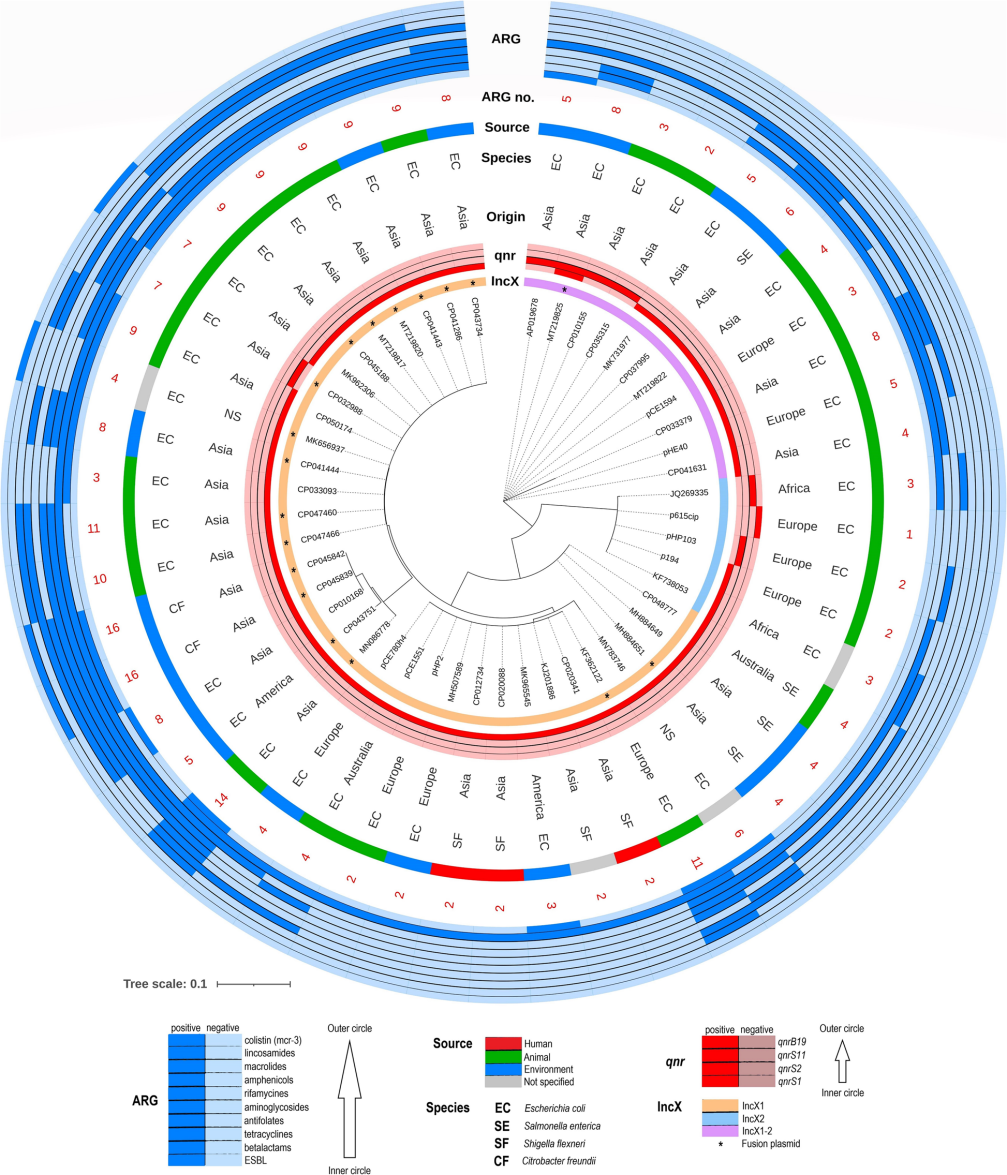
Phylogenetic tree constructed from IncX1 and IncX2 (hybrid and multi-replicon including) plasmids carrying *qnr* gene. ARG positivity represents the presence of antibiotic resistance genes encoding resistance to the mentioned groups of antibiotics. ARG no. represents the total number of antibiotic resistance genes for each featured plasmid. Plasmid with IncX region marked by * symbol represents multi-replicon plasmid carrying Col, FI, HI1, I, N or R replicons

The comprehensive comparison and phylogenetic analysis consisted of 49 plasmids, where 41 of them are publicly available from global collection and eight of them are presented in this study. The constructed phylogenetic tree was divided into three clusters (Fig. 7 and Supplementary Figure S4). IncX1 cluster (orange) was the most abundant (*n* = 33) while IncX2 cluster (blue) represented the minority (*n* = 5). The phylogenetic tree revealed a new hybrid IncX1-2 cluster (purple) consisting of 11 diverse plasmids. The phylogenetic analysis included 17 multi-replicon plasmids within the IncX1 cluster that carried other replicon types (Col, FI, HI1, I, N and R), resulting in high IncX1 group variability even in the plasmid backbone (Fig. 2).

Four variants of *qnr* gene were identified, *qnrS1* (*n* = 42)*, qnrS2* (*n* = 6)*, qnrS11* (*n* = 1) and *qnrB19* (*n* = 1) within the global collection of 49 IncX plasmids. Interestingly, plasmid pRW7-1_235k_tetX (MT219825) originating from a wastewater *E. coli* isolate from China carried both *qnrS1* and *qnrS2* variants. The most frequent variant of *qnr* gene associated with IncX1 and IncX2 plasmids was *qnrS1* (85.7%) followed by *qnrS2* (12.2%).

### Frequency of IncX plasmid transfer

Substantial differences in frequency of plasmid transfer in the IncX group ranging from 3.26 × 10^–7^ to 3.79 × 10^–2^ T/D were demonstrated (Table 1). Individual plasmids within the IncX1 and IncX2 groups showed similar frequencies while statistically significant differences (Supplementary Table S5) were observed between IncX1 and IncX2 group. The IncX1 group demonstrated a higher frequency of plasmid transfer compared to IncX2 and the hybrid variant.

The representative plasmids were selected for further experiments for each monitored group, pHP2 for IncX1 and p194 for IncX2 group as presented below. They belong to the most disseminated IncX lineages identified in our previous study [15]. The IncX1-2 plasmids were excluded from further experiments due to low nearly undetectable values of transfer rate obtained within the first part of experiments shown above.

**Fig. 8:**
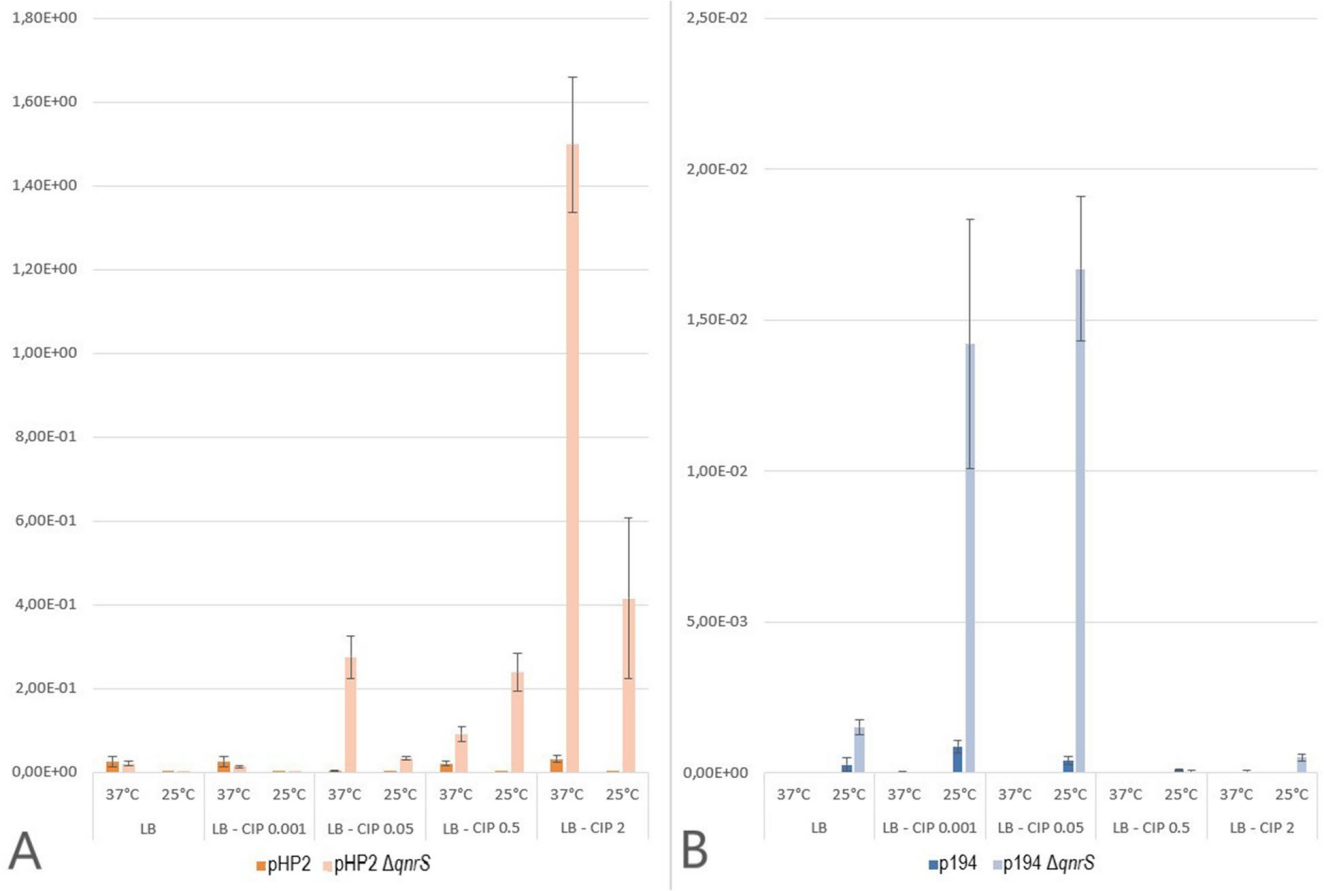
Influence of induced stress on IncX1 **A** and IncX2 **B** plasmid conjugative transfer. Frequency of transfer was calculated as the number of transconjugants per the number of donors (T/D). LB stands for Luria–Bertani agar. CIP represents the concentration (µg/mL) of supplemented ciprofloxacin into the media. Due to high differences in transfer frequencies the lower values are not clearly depicted. Exact values are specified in Table 2

Conjugation frequencies of pHP2 (IncX1) and p194 (IncX2) and their mutant variants (*qnrS* knockout—pHP2 *ΔqnrS* and p194 *ΔqnrS)*, were determined both without stress and under stress of temperature change and supplementation of various concentration of ciprofloxacin (Table 2, Fig. 8).

**Table 2:** Frequency of IncX plasmid conjugative transfer under induced stress

Media^a^	T^b^	IncX1 (pHP2)	Standard deviation	IncX1 (pHP2 *ΔqnrS*)	Standard deviation	IncX2 (p194)	Standard deviation	IncX2 (p194 *ΔqnrS*)	Standard deviation
**LB**	37 °C	2.56 × 10^–2^	1.26 × 10^–2^	2.12 × 10^–2^	5.29 × 10^–3^	1.34 × 10^–6^	4.92 × 10^–7^	6.83 × 10^–6^	2.53 × 10^–6^
	25 °C	1.45 × 10^–4^	5.95 × 10^–5^	3.84 × 10^–4^	9.71 × 10^–5^	2.77 × 10^–4^	2.25 × 10^–4^	1.52 × 10^–3^	2.41 × 10^–4^
**LB—CIP 0.001**	37 °C	2.63 × 10^–2^	1.22 × 10^–2^	1.39 × 10^–2^	2.85 × 10^–3^	3.47 × 10^–5^	3.24 × 10^–6^	7.09 × 10^–6^	2.63 × 10^–6^
	25 °C	4.85 × 10^–4^	4.24 × 10^–5^	2.27 × 10^–4^	6.19 × 10^–5^	8.77 × 10^–4^	2.05 × 10^–4^	1.42 × 10^–2^	4.11 × 10^–3^
**LB—CIP 0.05**	37 °C	3.34 × 10^–3^	1.19 × 10^–3^	2.76 × 10^–1^	5.06 × 10^–2^	1.32 × 10^–6^	1.56 × 10^–7^	1.34 × 10^–6^	7.78 × 10^–7^
	25 °C	4.25 × 10^–4^	5.34 × 10^–5^	3.43 × 10^–2^	4.96 × 10^–3^	4.01 × 10^–4^	1.24 × 10^–4^	1.67 × 10^–2^	2.40 × 10^–3^
**LB—CIP 0.5**	37 °C	2.11 × 10^–2^	4.70 × 10^–3^	9.21 × 10^–2^	1.82 × 10^–2^	2.52 × 10^–6^	1.94 × 10^–6^	4.10 × 10^–6^	2.18 × 10^–6^
	25 °C	1.17 × 10^–4^	3.13 × 10^–5^	2.38 × 10^–1^	4.51 × 10^–2^	9.23 × 10^–5^	2.30 × 10^–5^	4.55 × 10^–5^	5.65 × 10^–5^
**LB—CIP 2**	37 °C	3.12 × 10^–2^	8.22 × 10^–3^	1.50 × 10^–0^	1.61 × 10^–1^	4.07 × 10^–8^	7.04 × 10^–8^	4.45 × 10^–5^	3.08 × 10^–5^
	25 °C	2.71 × 10^–4^	3.71 × 10^–5^	4.16 × 10^–1^	1.92 × 10^–1^	1.37 × 10^–5^	2.37 × 10^–6^	5.14 × 10^–4^	1.24 × 10^–4^

Frequency of transfer was calculated as the number of transconjugants per the number of donors (T/D)

^**a**^**LB** stands for Luria–Bertani agar. **CIP** represents the concentration (µg/mL) of supplemented ciprofloxacin into the media

^**b**^**T** represents the incubation temperature during conjugation

### Combined stress factors reduce wildtype IncX1 transfer rate while single stress factors alter the IncX1 *ΔqnrS* transfer rate

Only a combination of temperature change and high concentration of supplemented ciprofloxacin significantly reduced the wildtype IncX1 transfer rate (Table 2, Fig. 8). The transfer rate of wildtype pHP2 was significantly reduced (*p* = 0.016 and 0.023) by lowering the mating temperature to 25 °C only in combination with supplementation of higher concentrations of ciprofloxacin (0.5 and 2 µg/mL, respectively). While neither temperature change nor ciprofloxacin supplementation as a singular stress factor on their own has a significant effect on the frequency of wildtype IncX1 plasmid transfer. The temperature change to 25 °C reduced the transfer rate but was not statistically significant, although it was close to the limit of significance (*p* = 0.073). The ciprofloxacin supplementation alone did not significantly affect wildtype IncX1 transfer with *p* = 0.671, 0.080, 0.460 and 0.652 at ciprofloxacin at concentrations 0.001, 0.05, 0.5 and 2 µg/mL, respectively.

The transfer of pHP2 *ΔqnrS* was significantly reduced by temperature change to 25 °C (*p* = 0.021). On the other hand, the pHP2 *ΔqnrS* transfer was significantly increased by stress caused by supplementation of ciprofloxacin alone at concentrations above 0.05 µg/mL (*p* = 0.016, 0.015 and 0.004, respectively). The temperature change to 25 °C significantly decreased (*p* < 0.015) the frequency of transfer also in all monitored concentrations with exception of 0.5 µg/mL (*p* = 0.014) in which the frequency of transfer increased.

The pHP2 *ΔqnrS* showed no significant differences in transfer compared to the wildtype pHP2 transfer at 37 °C without ciprofloxacin supplementation (*p* = 0.691). A significant increase in frequency of the pHP2 *ΔqnrS* transfer compared to the wild type pHP2 transfer occurred under the conditions of 37 °C in combination with increasing ciprofloxacin concentration (0.05, 0.5 and 2 µg/mL with *p* = 0.011, 0.032 and 0.004, respectively). At 25 °C, this significant increase in frequency started even in lower concentrations from 0.001 µg/mL (*p* = 0.007) and higher with the exception of concentration at 2 µg/mL which had no significant difference (*p* = 0.065). The frequency of pHP2 *ΔqnrS* transfer significantly increased with induced stress caused by supplementation of ciprofloxacin but, on the other hand, it was significantly reduced by temperature change to 25 °C. Therefore, these results demonstrated that disruption of *qnrS* gene may contribute to changes in plasmid transfer frequency under influence of induced stress.

### The frequency of wildtype IncX2 transfer increases with supplemented ciprofloxacin whereas the temperature change to 25 °C increased IncX2 *ΔqnrS* transfer

The transfer of wildtype IncX2 plasmid, p194, was not significantly affected by temperature change to 25 °C (*p* = 0.168). The supplementation of ciprofloxacin alone significantly altered the frequency of p194 transfer, specifically an increase at concentration 0.001 µg/mL (*p* = 0.002) and decrease at 2 µg/mL (*p* = 0.034). The transfer rate of wildtype p194 was significantly increased by lowering the mating temperature to 25 °C in combination with supplementation of any concentration of ciprofloxacin (0.001, 0.05, 0.5 and 2 µg/mL with *p* = 0.020, 0.031, 0.025 and 0.009, respectively). The transfer of wildtype IncX2 plasmid demonstrated exactly an opposite tendency compared to the wildtype IncX1 plasmid transfer, an increase in frequency at 25 °C with supplementation of ciprofloxacin.

In regard to IncX2 plasmid with *qnrS* gene knockout, p194 *ΔqnrS*, the frequency of transfer was significantly increased at 25 °C (*p* = 0.009). On the other hand, the supplementation of ciprofloxacin had no significant effect on frequency of p194 *ΔqnrS* transfer at 37 °C (*p* = 0.895, 0.075, 0.356 and 0.167 respectively). The transfer rate of p194 *ΔqnrS* was significantly increased at 25 °C with supplementation of ciprofloxacin in sub-inhibitory concentrations (c < 0.05 µg/mL) and with the highest concentration (2 µg/mL) compared to the p194 *ΔqnrS* transfer at 37 °C.

The p194 *ΔqnrS* showed no significant differences in transfer compared to the wildtype p194 transfer at 37 °C without ciprofloxacin supplementation (*p* = 0.066). The significant increase of p194 *ΔqnrS* transfer was observed at 25 °C without ciprofloxacin supplementation (*p* = 0.017). The increase in frequency of p194 *ΔqnrS* transfer at 37 °C was observed at 0.001 µg/mL ciprofloxacin (*p* = 0.001) while the decrease was detected at supplementation of 0.5 µg/mL (*p* = 0.022). Considering the combination of both induced stresses, the frequency of p194 *ΔqnrS* transfer compared to the wildtype p194 transfer was increased at 25 °C in the lowest and the highest concentration of supplemented ciprofloxacin (0.001 and 2 µg/mL with *p* = 0.019 and 0.019 respectively).

### Genetic background of bacteria affects the plasmid transfer

Conjugation transfer of wildtype IncX1 and IncX2 plasmids between closely and distantly related bacterial strains showed significant differences in relation to bacterial host as mating pairs (Table 3). The pBGC marker plasmid did not influence the frequency of transfer of monitored IncX plasmids as was observed in the comparison of transfers between *E. coli* TOP10 strain and *E. coli* A15 strain to *E. coli* TOP10 strain and *E. coli* A15 pBGC strain with no significant differences (*p* = 0.287).

**Table 3:** Influence of bacterial background on IncX plasmid transfer

Plasmid	Donor	Recipient	Frequency of transfer^a^	Standard deviation
pHP2 (IncX1)	*E. coli* TOP10 **ST10**	*E. coli* A15 pBGC **ST10**	2.36 × 10^–2^	6.32 × 10^–3^
		*E. coli* UPEC536 **ST127**	1.34 × 10^–6^	5.47 × 10^–7^
		*E. coli* UPEC536 pBGC **ST127**	6.81 × 10^–6^	5.60 × 10^–7^
		*E. coli* **ST131** pBGC	Not detected	Not detected
	*E. coli* UPEC536 **ST127**	*E. coli* UPEC536 pBGC **ST127**	1.01 × 10^–2^	1.01 × 10^–3^
p194 (IncX2)	*E. coli* TOP10 **ST10**	*E. coli* A15 pBGC **ST10**	1.04 × 10^–4^	2.39 × 10^–5^
		*E. coli* UPEC536 pBGC **ST127**	Not detected	Not detected
		*E. coli* **ST131** pBGC	Not detected	Not detected

^**a**^Frequency of transfer was calculated as the number of transconjugants per the number of donors (T/D)

The comparison of transfer rates under variable bacterial conditions indicated that frequency of transfer between cells with a similar taxonomic relatedness showed significantly higher transfer rate (Table 3). For IncX1 pHP2, the conjugative transfer from *E. coli* ST10 strain to another *E. coli* ST10 strain showed significantly higher frequencies than transfer from *E. coli* ST10 *to E. coli* ST127 strain with *p* = 0.023. Same tendencies were shown with transfer between *E. coli* ST127 strains. The transfer from *E. coli* ST127 strain to another *E. coli* ST127 strain was also significantly higher by four orders of magnitude (*p* = 0.003) compared to the transfer from *E. coli* ST10 to *E. coli* ST127 strain. Meanwhile, the frequencies of plasmid transfer between *E. coli* ST10 strains compared to transfer between *E. coli* ST127 strains were not significantly different (*p* = 0.079). The frequency of transfer of IncX2 plasmid, p194, showed the same tendencies as observed in pHP2. With regard to the lower frequency of IncX2 plasmid transfer compared to the IncX1, the transmission from *E. coli* ST10 to *E. coli* ST131 strain was not observed and therefore the second conjugation experiment between *E. coli* ST131 strains with p194 was not performed.

### Plasmids sustained within their hosts

Conducted experiments showed that IncX1 (pHP2, pHP2 *ΔqnrS*) and IncX2 (p194, p194 *ΔqnrS*) plasmids were able to persist within the host cell in the antibiotic-free environment for more than five consecutive days regardless the fact that the host cell lacked the need for plasmid to provide antibiotic resistance (data not shown). We also observed that the disruption of *qnrS* gene did not reduce plasmid stability and therefore did not affect its persistence within the cell.

## Discussion

### Phylogenetic analysis of IncX plasmids carrying *qnr* genes

Based on phylogenetic analysis the IncX1 plasmid group was the most abundant and included also multi-replicon plasmids among the IncX1 cluster. The IncX2 plasmid group exhibited the lowest occurrence which has previously been observed [36]. The hybrid IncX1-2 cluster including pHE40 and pCE1594 formed a remarkably large group compared to the other studies. So far, Guo et al*.* [37] (2017) described pYD786-3, the only previously observed IncX1-2 hybrid plasmid, while our study reveals wider representation of the IncX1-2 hybrid subtype carrying *qnr* genes. The limited publications of IncX1-2 plasmid hybrids may be due to their lower incidence or their complexity in detection and classification. We confirmed that the most frequent variant of *qnr* gene associated with IncX1 and IncX2 plasmids was *qnrS1* which has been observed also previously [38].

The IncX plasmids have a narrow-host range [29] and are not often observed in other bacteria outside Enterobacterales with the exception of *Pseudomonas* [15], which corresponds with the narrow range of bacterial hosts detected in our study including *E. coli* (*n* = 39), *S. enterica* and *S. flexneri* (both per 4) and *C. freundii* (*n* = 2). The IncX plasmids analysed here originated from bacteria in various niches including animals (*n* = 26), environment (*n* = 16), humans (*n* = 3) or of undetermined origin (*n* = 4). The dissemination of *qnr*-encoding IncX plasmids can be considered worldwide, since they were observed in each continent except Antarctica. The presence and possible transfer of PMQR poses a high risk to the animal and human antibiotic therapy, since PMQR in association with chromosomal mutations conferring quinolone resistance results in high-level quinolone resistance in *Enterobacteriaceae* [39]. Plasmids described in this study were most frequently of animal origin (53.1%) pointing out a possible zoonotic reservoir with potential of causing medical problems either via getting into the food chain or via occupational risk.

### Frequency of IncX plasmid transfer

Significant differences in frequency of conjugal transfer were detected where IncX1 group demonstrated higher frequency of plasmid transfer compared to IncX2 and IncX1-2 groups. Differences in transfer rates between IncX groups may have various causes. Liakopoulos et al*. *[40] (2018) suggested that transfer of IncX plasmids might be conserved for groups of the same origin and even proclaimed that plasmids of animal origin transferred more efficiently at 30 °C than human-derived plasmids. Another mechanism responsible for different plasmid conjugative transfer efficiency may be the quorum sensing or sensing of environmental conditions with regulatory systems activating the *tra* genes [41]. Lower rate of IncX2 plasmid transfer may also be the cause of lower occurrence of these plasmids in the environment however, this statement requires further investigation to determine the cause.

It is speculated that induced stress might cause cellular responses such as alterations of cell wall or changes in regulation of gene expression that consequentially increases plasmid transfer rate [42–45]. Our study showed substantial differences in frequency tendencies under variable stress conditions between the individual IncX plasmid groups. Significant differences in transfer were observed under the influence of induced stress. In a study by Lopatkin et al*.* (2016), a selection pressure or stress such as non-inhibitory levels of several antibiotics were applied to cells during conjugation. The selection pressure or induced stress also consequently did not play a role solely in conjugative transfer [46]. Also, in a study by Pallares-Vega et al*.* (2021), the IncP-1 plasmid conjugative transfer was significantly reduced by decreasing the mating temperature [11]. We followed up on their findings and expanded it with more insights using combined induced stresses and the knockout of *qnrS* gene, thus achieved diverse results under specific conditions during conjugation. We observed that the *qnrS* gene knockout alone did not significantly alter the plasmid transfer. Supplementation of sub-inhibitory concentrations of ciprofloxacin and levofloxacin has also been observed to induce the plasmid RP4 (IncP) conjugation from *E. coli* to *Pseudomonas aeruginosa* via stimulation of expression of *tra* genes [47]. Shun-Mei et al*.* (2018) also noted that the conjugation efficiency is not linked to the expression of genes associated with SOS response that is generally induced by supplementation of quinolones [48]. The mechanism of plasmid transfer modification is still not fully elucidated and requires further investigation.

### Influence of genetic background on plasmid transfer

The plasmid transfer is not dependent just on a plasmid itself but the background of bacterial hosts plays its crucial part as well [49]. Alderliesten et al*. *[50] (2020) proclaimed that taxonomic relatedness affects only the conjugation in liquid mating but we propose its effect on solid mating bacteria that is supported by Sheppard, Beddis, and Barraclough [49] (2020).

Our results suggested that a similar genetic background of the bacterial strain can facilitate the mobility of plasmids as was also observed by Dimitriu et al. [12] (2019). Their study strongly suggested the influence of restriction-modification systems in recipients and their contribution to biasing the plasmid transfer frequency. Two strains of *E. coli* ST10 were more inclined to conjugate since they had similar genetic background, and the same was detected between two *E. coli* ST127 strains. On the other hand, the transfer from *E. coli* ST10 to *E. coli* ST127 was distinctly lower. Considering the transfer to *E. coli* ST131, the low rates of transfer may not have been experimentally observed due to the limits of detection. Despite the low frequency, once the plasmid transfer occurred between distantly related bacteria, the frequency between further closely related strains stabilized at higher rates similar to the transfer between original closely related strains. This finding highlighted the possibility that once a plasmid crosses the borders of distantly related bacterial strains, it may enable a rapid spread between closely related and even pathogenic bacteria possibly by bypassing the restriction barrier.

### IncX plasmid persistence

Plasmid persistence within cells without the selective pressure was similarly observed in IncN plasmids carrying *qnrS1* or *qnrB2* gene in a study conducted by Segura et al*. *[51] (2020). Monitored IncN plasmids persisted within uropathogenic cells even without the selective pressure of fluoroquinolones. There are numerous reasons for plasmid persistence including partition and toxin/antitoxin systems. All of our IncX1 and IncX1-2 monitored plasmids carried *stbE* and *stbD* genes encoding a functional toxin/antitoxin system as described before by Unterholzner et al*.* [52] (2013). The absence of a toxin/antitoxin system on IncX2 plasmids may be one of the possible reasons for their low occurrence due to their lower stability within bacteria. However, this hypothesis was not confirmed by our study since the IncX1 and IncX2 plasmids persisted in all of the monitored cells. One of possible answers to plasmid stability was proposed by Carroll and Wong [53] (2018) who stated that high plasmid transfer rates may be the reason behind plasmid persistence. According to the numerical analysis model the horizontal plasmid transfer keeps the plasmid stability at balance [54]. Another possibility is that the plasmid burden is decreased by compensatory mutations in plasmid or chromosomal genes [55], or a proposed positive epistatic effect [56] which consequently minimizes the advantages of losing the plasmid and therefore the plasmid withdrawal does not occur. Gama et al*.* [57] (2020) analysed several mathematical models including multiple plasmids and noted that plasmid interactions affecting host fitness, conjugation rate or plasmid missegregation significantly influenced, most dominantly promoted, the plasmid persistence as well.

## Conclusion

This study showed the significant properties and behaviour of IncX plasmids carrying antibiotic resistance genes that are likely to play a role in their dissemination and stability in bacterial populations.

It demonstrated the hitherto unknown occurrence of IncX1-2 plasmids harbouring PMQR genes. However, IncX1-2 plasmid importance was impaired by its much lower transfer rate compared to IncX1 and IncX2 group. A knockout of the *qnr* gene was observed to modify the frequency of plasmid transfer under influence of induced stress. The influence of both stresses was needed to affect the transfer rate of wildtype IncX plasmids, whereas a single stress was sufficient to affect the IncX *ΔqnrS* plasmid transfer rate. These findings propose the stimulating effect of ciprofloxacin supplementation on the plasmid transfer that can be nullified by the carriage of a single PMQR gene. Our study also indicated that the frequency of transfer was biased towards the host phylogenetic proximity as the transfer between phylogenetically closely related bacterial strains was higher than between phylogenetically distantly related.

## Supplementary Information

**Additional file 1.** 

**Additional file 2: Supplementary Table S1. **Experimental design of individual mating assayswith induced stress using pHP2 (IncX1) and p194 (IncX2 

**Additional file 3:** **Supplementary Table S2. **Bacterial strains used formating assays

**Additional file 4:** **Supplementary Table S3. **Experimental design ofindividual mating assays with pHP2 (IncX1) and p194 (IncX2) using variousbacterial background

**Additional file 5: Supplementary Table S5. **Student's T-test of IncX-typeplasmid conjugative transfer 

## Data Availability

The datasets generated and/or analysed during the current study are available in the GenBank repository (see Table 1).
