# Supplementary figures and images for: Genomic analysis of qnr-harbouring IncX plasmids and their transferability within different hosts under induced stress
Source: BMC Microbiol. 2022 May 19;22:136. doi: 10.1186/s12866-022-02546-6 (PMC9118779; doi:10.1186/s12866-022-02546-6)

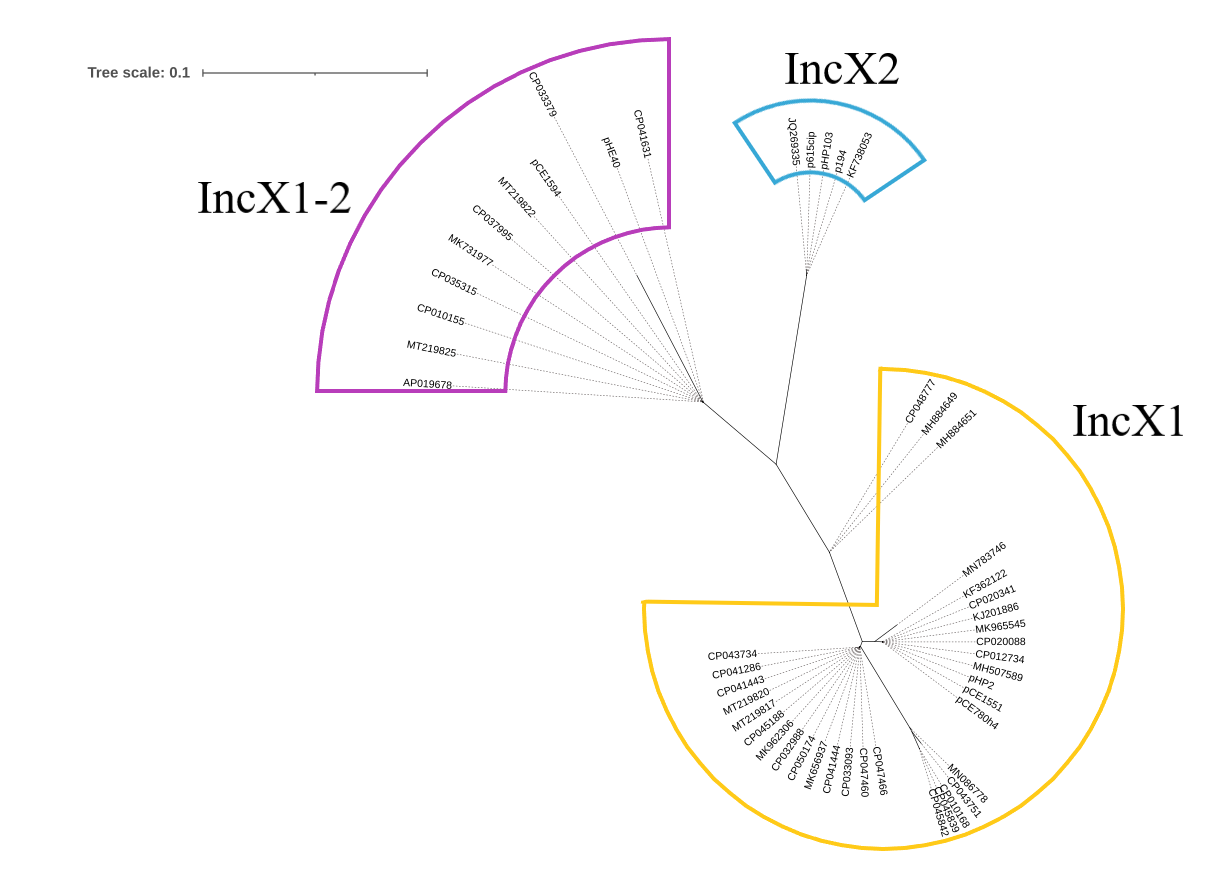

Supplement: Supplementary file 1 — Additional file 1. [file 12866_2022_2546_MOESM1_ESM.png]
