# Supplementary material for: Genomic analysis of qnr-harbouring IncX plasmids and their transferability within different hosts under induced stress
Source: BMC Microbiol. 2022 May 19;22:136. doi: 10.1186/s12866-022-02546-6 (PMC9118779; doi:10.1186/s12866-022-02546-6)
Supplement: Supplementary file 2 — Additional file 2: Supplementary Table S1. Experimental design of individual mating assayswith induced stress using pHP2 (IncX1) and p194 (IncX2 [file 12866_2022_2546_MOESM2_ESM.docx]

**Supplementary Table S1** Experimental design of individual mating assays with induced stress using pHP2 (IncX1) and p194 (IncX2)

| **Plasmid** | **Donor strain/Selection marker** | **Recipient strain/Selection marker** | **Transconjugant/Selection marker** |
| --- | --- | --- | --- |
| pHP2 | *Escherichia coli* TOP10 pHP2/Ampicillin (100 µg/mL) | *Escherichia coli* A15/Sodium azide (100 µg/mL) | *Escherichia coli* A15 pHP2/Ampicillin (100 µg/mL), Sodium azide (100 µg/mL) |
| pHP2 *ΔqnrS* | *Escherichia coli* TOP10 pHP2 *ΔqnrS/*Ampicillin (100 µg/mL) | *Escherichia coli* A15/Sodium azide (100 µg/mL) | *Escherichia coli* A15 pHP2 *ΔqnrS*/Ampicillin (100 µg/mL), Sodium azide (100 µg/mL) |
| p194 | *Escherichia coli* TOP10 p194/Tetracycline (20 µg/mL) | *Escherichia coli* A15/Sodium azide (100 µg/mL) | *Escherichia coli* A15 p194/Tetracycline (20 µg/mL), Sodium azide (100 µg/mL) |
| p194 *ΔqnrS* | *Escherichia coli* TOP10 p194 *ΔqnrS*/Tetracycline (20 µg/mL), | *Escherichia coli* A15/Sodium azide (100 µg/mL) | *Escherichia coli* A15 p194 *ΔqnrS*/Tetracycline (20 µg/mL), Sodium azide (100 µg/mL) |

Mentioned antibiotics were used for detection of particular donor, recipient or transconjugant colonies on LB agar plates.
