# Supplementary material for: Genomic analysis of qnr-harbouring IncX plasmids and their transferability within different hosts under induced stress
Source: BMC Microbiol. 2022 May 19;22:136. doi: 10.1186/s12866-022-02546-6 (PMC9118779; doi:10.1186/s12866-022-02546-6)
Supplement: Supplementary file 3 — Additional file 3: Supplementary Table S2. Bacterial strains used formating assays [file 12866_2022_2546_MOESM3_ESM.docx]

**Supplementary Table S2** Bacterial strains used for mating assays

| **Bacterial strain** | **Compound/antibiotic – concentration (µg/mL)** | **Sequence type** | **Reference** |
| --- | --- | --- | --- |
| *Escherichia coli* TOP10 | - | ST10 | Invitrogen^TM^, USA |
| *Escherichia coli* A15 | Sodium azide – 100 | ST10 | Faculty of Medicine in Pilsen, Charles University, CZ |
| *Escherichia coli* A15 pBGC | Sodium azide – 100, Chloramphenicol – 30 | ST10 | This study |
| *Escherichia coli* UPEC536 | Rifampicin – 25, Sodium azide – 100 | ST127 | Dept. of Clinical Science, University of Bergen, Norway, CP000247 |
| *Escherichia coli* UPEC536 pBGC | Rifampicin – 25, Sodium azide – 100 Chloramphenicol – 30 | ST127 | This study |
| *Escherichia coli* ST131 pBGC | Rifampicin – 25, Sodium azide – 100 Chloramphenicol – 30 | ST131 | CEITEC UVPS, CZ; marked with plasmid pBGC in this study |
