# Supplementary material for: Genomic analysis of qnr-harbouring IncX plasmids and their transferability within different hosts under induced stress
Source: BMC Microbiol. 2022 May 19;22:136. doi: 10.1186/s12866-022-02546-6 (PMC9118779; doi:10.1186/s12866-022-02546-6)
Supplement: Supplementary file 4 — Additional file 4: Supplementary Table S3. Experimental design ofindividual mating assays with pHP2 (IncX1) and p194 (IncX2) using variousbacterial background [file 12866_2022_2546_MOESM4_ESM.docx]

**Supplementary Table S3** Experimental design of individual mating assays with pHP2 (IncX1) and p194 (IncX2) using various bacterial background

| **Transfer type** | **Plasmid** | **Donor strain** | **Recipient strain** | **Transconjugant** |
| --- | --- | --- | --- | --- |
| Closely related | pHP2 | *Escherichia coli* TOP10 **ST10** pHP2 – Ampicillin (100 µg/mL) | *Escherichia coli* A15 **ST10** pBGC – Chloramphenicol (30 µg/mL) | *Escherichia coli* A15 pBGC pHP2 – Ampicillin (100 µg/mL), Chloramphenicol (30 µg/mL) |
| Distantly related | pHP2 | *Escherichia coli* TOP10 **ST10** pHP2 – Ampicillin (100 µg/mL) | *Escherichia coli* UPEC536 **ST127** – Rifampicin (25 µg/mL) | *Escherichia coli* UPEC536 pHP2 – Ampicillin (100 µg/mL), Rifampicin (25 µg/mL) |
| Distantly related | pHP2 | *Escherichia coli* TOP10 **ST10** pHP2 – Ampicillin (100 µg/mL) | *Escherichia coli* UPEC536 **ST127** pBGC – Chloramphenicol (30 µg/mL) | *Escherichia coli* UPEC536 pBGC pHP2 – Ampicillin (100 µg/mL), Chloramphenicol (30 µg/mL) |
| Distantly related | pHP2 | *Escherichia coli* TOP10 **ST10** pHP2 – Ampicillin (100 µg/mL) | *Escherichia coli* **ST131** pBGC – Chloramphenicol (30 µg/mL) | *Escherichia coli* ST131 pBGC pHP2 – Ampicillin (100 µg/mL), Chloramphenicol (30 µg/mL) |
| Closely related | pHP2 | *Escherichia coli* UPEC536 **ST127** pHP2 – Ampicillin (100 µg/mL), Rifampicin (25 µg/mL) | *Escherichia coli* UPEC536 **ST127** pBGC – Chloramphenicol (30 µg/mL) | *Escherichia coli* UPEC536 pBGC pHP2 – Ampicillin (100 µg/mL), Chloramphenicol (30 µg/mL) |
| Closely related | p194 | *Escherichia coli* TOP10 **ST10** p194 – Tetracycline (20 µg/mL) | *Escherichia coli* A15 **ST10** pBGC – Chloramphenicol (30 µg/mL) | *Escherichia coli* A15 pBGC p194 – Tetracycline (20 µg/mL), Chloramphenicol (30 µg/mL) |
| Closely related | p194 | *Escherichia coli* TOP10 **ST10** p194 – Tetracycline (20 µg/mL) | *Escherichia coli* UPEC536 **ST127** pBGC – Chloramphenicol (30 µg/mL) | *Escherichia coli* UPEC536 pBGC p194 – Tetracycline (20 µg/mL), Chloramphenicol (30 µg/mL) |
| Distantly related | p194 | *Escherichia coli* TOP10 **ST10** p194 – Tetracycline (20 µg/mL) | *Escherichia coli* **ST131** pBGC – Chloramphenicol (30 µg/mL) | *Escherichia coli* ST131 pBGC p194 – Tetracycline (20 µg/mL), Chloramphenicol (30 µg/mL) |

Mentioned antibiotics were used for detection of particular donor, recipient or transconjugant colonies on LB agar plates.

Closely related transfer type is missing for plasmid p194 due to Distantly related mating assays with frequency of transfer below detection limit and therefore the absence of specific donor for Closely related transfer.
