# Supplementary material for: Genomic analysis of qnr-harbouring IncX plasmids and their transferability within different hosts under induced stress
Source: BMC Microbiol. 2022 May 19;22:136. doi: 10.1186/s12866-022-02546-6 (PMC9118779; doi:10.1186/s12866-022-02546-6)
Supplement: Supplementary file 5 — Additional file 5: Supplementary Table S5. Student's T-test of IncX-typeplasmid conjugative transfer [file 12866_2022_2546_MOESM5_ESM.docx]

**Supplementary Table S5** Student's T-test of IncX-type plasmid conjugative transfer.

|  |  | **IncX1** | | | **IncX1-2** | | **IncX2** | | |
| --- | --- | --- | --- | --- | --- | --- | --- | --- | --- |
|  | **FoT** | **pHP2** | **pCE780h4** | **pCE1551** | **pHE40** | **pCE1594** | **p194** | **p615cip** | **pHE103** |
| **IncX1** | **pHP2** |  | 0.16606 | 0.10751 | 0.02195 | 0.02195 | 0.02207 | 0.02145 | 0.02171 |
|  | **pCE780h4** |  |  | 0.88684 | 0.00865 | 0.00865 | 0.00869 | 0.00906 | 0.00929 |
|  | **pCE1551** |  |  |  | 0.03662 | 0.03663 | 0.03685 | 0.03771 | 0.03632 |
| **IncX1-2** | **pHE40** |  |  |  |  | 0.85997 | 0.01713 | 0.01722 | 0.07509 |
|  | **pCE1594** |  |  |  |  |  | 0.15162 | 0.15168 | 0.07521 |
| **IncX2** | **p194** |  |  |  |  |  |  | 0.19314 | 0.14752 |
|  | **p615cip** |  |  |  |  |  |  |  | 0.59275 |
|  | **pHE103** |  |  |  |  |  |  |  |  |

The green values (p value < 0.05) represent significant differences between the frequency of transfer (FoT) of IncX plasmids within individual groups while the red value represent their similarity.
